# Overdone Advocacy *v*. Common Sense

**Published:** 1918-06-29

**Authors:** 


### THE AGITATION FOR UNQUALIFIED PRACTITIONERS.

### Overdone Advocacy v. Common Sense.

That part of the public press that has lent itself to the agitation to get the Government to give countenance to unregistered practitioners of surgery and medicine has been announcing, with a sort of air of triumph, that no obstacle is to be placed in the way of an officer or man who wishes to consult such. Almost it might be imagined that formerly such obstacles existed. In fact, no one in the whole of Great Britain or Ireland is precluded from consulting any unregistered man or woman about matters of health provided they are prepared to satisfy the demands of the person they consult. The demands will usually include payment in advance because the law of the land declines to allow the courts of the land to enforce a demand for payment for medical or surgical treatment or advice made by an unregistered practitioner. One uninstructed reading, say, the voluminous and heated dissertations of a certain reverend gentleman and others that have appeared in the *Nineteenth Century*, and certain articles that have appeared in *Truth*, might imagine a great conspiracy of a mighty trade union to suppress new knowledge and, in its own mean interests, to keep healing from suffering mankind.

In the cold light of common sense what is the case? In the first case, and most unfortunately in many people's opinion, there is no medical trade union. It has been urged on the profession and has not been adopted. The law of the land allows anyone in the land to practise healing either by medical or by surgical means and to advertise that he or she does so. The only obstacles are that no one must represent that he or she is a duly qualified and registered medical practitioner if not so, no one may sign a death certificate or certain other certificates who is not registered, and the courts will not take action to enforce the payment of fees demanded by the unregistered. There is no objection to the unregistered refusing to give treatment or advice until a fee has been paid. On this last point the unregistered practitioner has great advantage over the registered, for the latter is always liable to censure by a coroner's jury if he refuses to attend unless a fee is paid in advance, and the patient who has not found the fee should die untended. The unregistered is never liable to this usually quite unjust censure.

Because there were obvious drawbacks to unlimited medical practice with nothing to distinguish those who had taken some trouble to attain a fair knowledge of the art of healing from those who had not, the representatives of the people in Parliament assembled took action to regulate medical practice. This was not done in the interests of the doctors and surgeons but of the general public. Men, and later women, who had acquired a certain standard of knowledge were put on a register. To obtain admission to the register it was made necessary that the candidates should satisfy the authorities of certain universities, Oxford, Cambridge, Dublin, Edinburgh, etc., or of certain bodies corporate, such as the Royal Colleges of Surgeons and Physicians, the Society of Apothecaries, etc., that he had a certain amount of knowledge. The mode of ascertaining and satisfying themselves that the candidate had at least the minimum of knowledge on which it was desirable he should be allowed to practise on the public with the *imprimatur* of the registering authority adopted by the universities and bodies corporate is, and has been, a mixture of examination and certificate of diligent attendance to studies. On the whole the result has been satisfactory. Men grossly ignorant cannot get on to the register. Of course men on the register who take no pains to retain and to improve their knowledge of their profession can become dangerously ignorant of it, but on the whole the necessity of making a living against competition and the real love of his work which is in most of the men who follow scientific professions keeps up the level.

The General Medical Council makes rules to regulate the conduct of the registered towards one another and towards the general public, and enforces obedience by its power of removing from the register for conduct not approved.

These regulations are every one made as much or more for the advantage of the public as for the advantage of the registered practitioner. For instance, if a registered man has improper relations with a female patient he is "struck off" as not being worthy of trust. If a man performs an operation to procure abortion and is convicted of it in a court of law he is struck off. The first case is to prevent the public being subjected, under the sanction of the medical authority, to the attentions of such men. The second case is because the man is a felon by British law, and therefore is not to be recognised as a member of an honourable and authorised company of men and women.

Always, in every body of men, there will be those who, for private gain, will seek to evade the spirit of regulations. The object of some of the regulations was to prevent the public being treated, without its knowledge, by men who had not, by steady, regular, and diligent studies, attained skill in the practice of medicine and surgery. Such men as had qualified and registered, because the number of them was limited, and the capital invested by them in gaining their knowledge large, were able to command a fairly high wage as assistants to men established in practice. Some men in practice found gain by employing men not qualified and registered to work for them because they were cheaper. These were the unqualified assistants. They became a considerable body. Often they were clever both in diagnosis

**Agitation for Unqualified Practitioners**—(*continued*).

and treatment; most worthy men who had by long experience acquired considerable skill. So much had they become a recognised if irregular adjunct to practice that most of those who employed them did not recognise any wrong in so doing, and certainly by the poorer patients of a large general practice they were well received. The question of qualification or registration was seldom raised. The writer knew several such of varying skill, but all excellent, hard-working men, though some were not to be trusted with alcohol. In fact many were men who, by reason of drinking habits, had failed successfully to complete their studies. Under honest principals who used them reasonably they did useful work.

But if irregularities are not stopped they tend to grow greater. Registered men started branch practices by establishing in a surgery an unqualified man to whom they paid so much a year and made handsome profits, the balance of their assistants' earnings. When a patient was dangerously ill or seemed like to die, the principal was sent for so that he could sign the death certificate if necessary. Parallel with this was another practice. Any man of any degree of ignorance would advertise himself as a curer and start a practice. To avoid difficulties with the law he would arrange with some unscrupulous medical man on the register to sign death certificates for him. "Covering" had arisen and become a scandal. All this had to be stopped; not in the interests of the doctors but in the interests of the public. The rule was made—No registered practitioner may practise with nor assist in the practice of an unregistered practitioner. It is a perfectly sound rule and is rightly enforced.

No one claims that an unregistered man may not be in every or any branch of medicine or surgery the equal of any registered man, but as a rule the irregular practitioner is not a skilled man nor safe to trust. There is no question that there have been men of great skill amongst the irregular, unregistered, advertising practitioners.

Of the unregistered, one branch was the bone-setters. Many have flourished; some with more than a local reputation. It was to this branch that Mr. Barker, around whose name so much writing has been written, belonged. He especially has devoted himself to the treatment of injured joints. Why he did not study surgery in the ordinary ways, present himself for examination and go on the register, is not known. He did not do so, though it is probable that to a man of his ability ordinary qualification would have been easy. It is possible that he was shrewd enough to see that the opportunity for advertisement that keeping off the register would give him was an advantage to his pocket. Whatever his reasons, he did not see fit to register, and therefore finds that men on the register cannot associate themselves with him in practice because their rules forbid it. Of course it would be possible to pass a special regulation of the General Medical Council exempting Mr. Barker from the incidence of the rule, but if that were done where are the exceptions to stop? There are other unregistered men as able as Mr. Barker, if not in his branch, in other branches. They would have a grievance if Mr. Barker was excepted from amongst them. It might come to a special examination to decide who amongst the unregistered should be associated with the registered.

Then the question of advertising comes in. It was thought better for the public and for the advancement of medicine that medical men should not be allowed to advertise. The reasons for this course are so obvious that it is not worth while to detail them. The unregistered man is free to advertise himself and his cure in any fashion he likes. If a registered man is allowed to associate himself professionally with the unregistered, he will gain an advantage of advertisement which his *confrère* who has no unregistered colleague will lack. It follows that every doctor must be allowed to advertise or there will be a handicap in favour of a few.

One of the reasons for forbidding advertisement was because it was thought that the prohibition would spread medical discoveries. If a man could advertise that he and he only had discovered the cure for some disease, he would do so, and seek to prevent the nature of that cure leaking out so that he might have the pecuniary advantages of his secret and hidden knowledge. Since he may not do that, he makes his discovery, or fancied discovery, known to the profession at large by means of a book, a pamphlet, or an article, and so, if his work has been good, attains honour and increase of practice. The new discovery is scrutinised by hundreds of able men, improved upon or discarded or adopted as it is as the case may be. It is open to any man in the world who thinks he has a surgical process that would be to the advantage of humanity if it were widely known, to publish a book describing it and to illustrate the book with selected cases. It is scarcely fair to abuse a profession, the members of which publish their discoveries so that all may benefit, because the profession does not go hat in hand to ask to have special instruction in a method which could be made known in the usual way. It cannot be a wise policy to open doors which shall admit to official recognition men who have not been through the special courses of study laid down as necessary to competence and who have only the proof of common report or of bold advertisement that they have any competence at all. No matter how good a pleader he may be, a man cannot conduct a case in court if he has not been called to the Bar. No matter how good a man or how excellent a preacher he may be, a man cannot take a service in a parish church if he be not properly ordained. And no barrister will associate himself professionally with such as the first case, and no parson of the Church of England consider for a moment associating himself with such as the second. Why, then, should lawyers and parsons write as if the medical profession was vile, unscrupulous, and selfish because it stands by analogous regulations?

